# From small molecules to polymeric probes: recent advancements of formaldehyde sensors

**DOI:** 10.1080/14686996.2021.2018920

**Published:** 2022-02-14

**Authors:** Swagata Pan, Subhadip Roy, Neha Choudhury, Priyanka Priyadarshini Behera, Kannan Sivaprakasam, Latha Ramakrishnan, Priyadarsi De

**Affiliations:** a Polymer Research Centre, Department of Chemical Sciences, Indian Institute of Science Education and Research Kolkata, Mohanpur, India; b Centre for Advanced Functional Materials, Department of Chemical Sciences, Indian Institute of Science Education and Research Kolkata, Mohanpur, India; c Department of Chemistry and Biochemistry, St. Cloud State University, Saint Cloud, MN, USA; d College of Science and Technology, Bloomsburg University, Bloomsburg, PA, USA

**Keywords:** Formaldehyde, sensors, polymer, fluorophore, sensing mechanism, 20 Organic and soft materials (colloids, liquid crystals, gel, polymers), 208 Sensors and actuators < 200 Applications

## Abstract

Formaldehyde is a well-known industrial material regularly used in fishery, vegetable markets, and fruit shops for maintaining their freshness. But due to its carcinogenic nature and other toxic effects, it is very important to detect it in very low concentrations. In recent years, amine-containing fluorescent probes have gained significant attention for designing formaldehyde sensors. However, the major drawbacks of these small molecular probes are low sensitivity and long exposure time, which limits their real-life applications. In this regard, polymeric probes have gained significant attention to overcome the aforementioned problems. Several polymeric probes have been utilized as a coating material, nanoparticle, quartz crystal microbalance (QCM), etc., for the selective and sensitive detection of formaldehyde. The main objective of this review article is to comprehensively describe the recent advancements in formaldehyde sensors based on small molecules and polymers, and their successful applications in various fields, especially in situ formaldehyde sensing in biological systems.

## Graphical abstract

**Figure:**
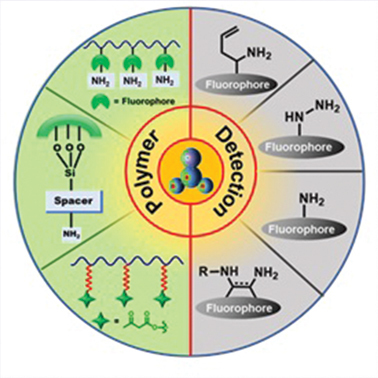

## 1. Introduction

**Figure 1.**
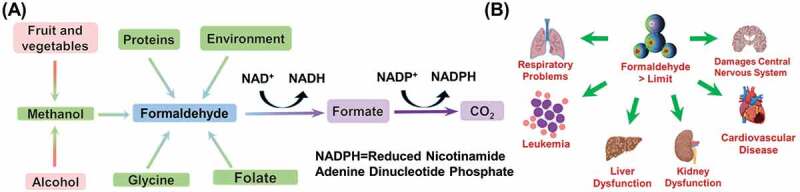
(a) Formation of Formaldehyde in the body, and (b) Formaldehyde’s harmful effects.

Formaldehyde (FA, HCHO), is the simplest aldehyde, and its 40% aqueous solution is known as formalin. It is a colorless, poisonous gas with a strong smell, that causes severe damage to our central nervous system (CNS) and immune system [1]. It is generally used in the making of many resins, polymers, wood products [2], and protein cross-linkers for tissue fixation [3]. The thermal and chemical decomposition of construction materials such as particleboard and urea-formaldehyde foam insulation heavily release FA in the atmosphere [1]. FA is well known for its toxicity and carcinogenic nature [4]. In humans, formaldehyde transforms into formic acid, giving rise to breathing difficulties, hypothermia, blood acidity, and coma or death [5]. Recently, International Agency for Research on Cancer (IARC) has announced formaldehyde as one of the main Group 1 carcinogenic organic compounds in the World [6]. The World Health Organization (WHO) declared the limit of exposure to formaldehyde as 80 parts per billion (ppb) as standard for 30 min [7]. Occupational Safety and Health Administration (OSHA) fixed standard value for permissible exposure limit at 750 ppb, while maximum exposure value for Immediately Dangerous to Life or Health (IDLH) is considered as 20 parts per million (ppm) [8,9]. US Environmental Protection Agency has determined the maximum reference dosage of FA is 0.15–0.2 mg/kg body weight/day [10]. In the brains of healthy individuals, the concentration of FA is in the 0.2–0.4 mM range [11]. In body tissues, FA reacts with biomolecules like amino acids, proteins, nucleic acids and nucleotides, and produces unstable assemblies which transport FA to distant tissues from the respiratory tract [12]. Consumed FA causes damage in liver and kidney (Figure 1b), which leads to albuminuria, jaundice, haematuria and acidosis, anuria, or inactivity in central nervous system leading to depression, and even causing heart failure [5,13]. Even a low level of FA harms the respiratory organs, nose, eyes, and originates allergies, commonly identified as sick house syndrome [14]. Ingestion of 30 mL of 37% HCHO solution is reported to cause death in humans [15]. Moreover, FA is toxic to neuronal cells and is able to disrupt the neuronal networks through *τ* protein polymerization and induction of hyperphosphorylation [16].

Due to the fact that it is highly dangerous for mankind and nature, it has become very important to detect formaldehyde at a low concentration level. Nowadays many techniques are used to detect it efficiently, such as Raman spectroscopy [17], liquid chromatography [18], gas chromatography [19], sol-gel methods [20], transmission electron microscopy (TEM) [21], conductometry [22,23], biosensor methods [24], and ion chromatography [25]. These techniques are highly sensitive, but not suitable for the detection of FA in living cells. In contrast, fluorescent probes have attracted significant attention from researchers due to their facile detection along with their highly sensitive nature. Consequently, in recent years researchers have developed several efficient fluorescent sensors for FA by taking the benefit of the aza-Cope rearrangement [26,27] or formaldehyde amine condensation [28,29]. Yet, the probes used for detection usually take a long response time due to the slow reaction between the probe and formaldehyde. Most of the reported probes are usually water-insoluble, thus some amount of volatile organic solvents is added into the aqueous media, which restricts their biological application. Also, the probes are not biodegradable and hence there remains a challenge of excretion from our body. In this regard, polymeric probes have emerged as an optimal solution to the above-mentioned problems.

Polymeric probes are being used in several sensing [30,31] and real-life applications to overcome the problems faced by small molecular probes. Recently, our group has published several articles on polymeric sensors [32,33] for the selective detection of metals [34,35] and volatile organic compounds [36,37]. Thus, hydrophilic fluorescent polymeric sensors with pendant primary amine groups have emerged as an alternative approach for FA sensing as they offer improved water solubility and high sensitivity. Various functional moieties are generally incorporated into the side chain or polymeric backbone to design polymeric sensing materials [38,39]. In this regard, polymeric probes are considered as an outstanding platform due to their multiple recognition sites that can bind with the lower concentration of analyte more effectively, and result in faster fluorescence response as well as desirable signal amplification [40,41]. Even though various polymeric sensors have been successfully developed for numerous environmental or food pollutants, very less effort has been devoted to fabricate water-soluble, fast-response polymeric materials for sensing formaldehyde [42,43]. Therefore, a detailed investigation in this regard is necessary to obtain a thorough understanding to design effective fast-response polymeric sensors having good aqueous solubility. Although some review articles have been published [44,45] focusing on the small molecular probes, to the best of our knowledge, there is no review article accentuating the importance of polymeric materials in FA sensing. In this review article, our major aim is to comprehensively review different types of reaction-based techniques for FA sensing, and their successful applications in various fields, especially *in situ* formaldehyde sensing.

## 2. Detection of FA using small molecule-based sensors

Fluorescent imaging has become a crucial part of recent scientific discoveries due to its ability to observe changes inside the cells, and tissues. Consequently, scientists have used this method of fluorescence detection for rapid and accurate detection of FA. According to recent research, in most cases of formaldehyde-detecting fluorescent probes, the amino group of the probe and the carbonyl group of HCHO generally react to detect formaldehyde *via* colorimetric changes. The detection-based reactions are generally categorized into two classes: (1) formimine type of intermediate forms from the reaction between FA and primary amine of probes, this consists of three types (i) 2-aza-Cope rearrangement [46,47] and subsequent *β*-elimination from the probe [48,49]; (ii) Schiff base-based probe [50,51]; (iii) aromatic hydrazine [52,53], and (2) aminal formation *via* reaction of FA and *o*-diamino probes [54,55]. Using these reactions and by their functional modifications, FA sensing mechanism can be attained *via* different processes, like photoinduced electron transfer (PET) [56,57], spiro cyclization [58], intramolecular charge transfer (ICT) [59], and also different response mechanisms such as, ratiometric [60] and fluorophore turn-on-based detection [61]. Also, many other types of reactions such as cyclization [62], ring-opening reaction [63], and nucleophilic addition [64] are used to detect FA.

### 2.1. 2-Aza-Cope rearrangement-based fluorescent probe

**Figure 2.**
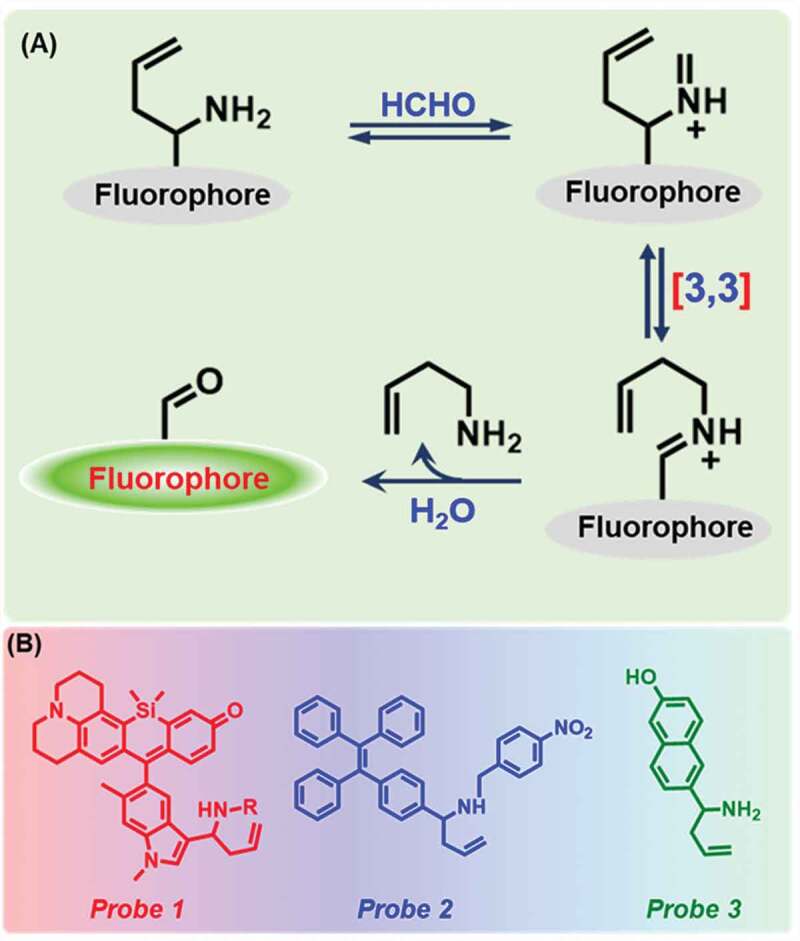
Schematic representation of 2-aza-Cope rearrangement reaction and chemical structures of probes **1, 2**, and **3.**

Aza-Cope rearrangement reaction is currently used as a successful method to detect FA [26]. If an amine group, more specifically a homoallylic one bearing a fluorescent probe, reacts with formaldehyde, 2-aza-Cope sigmatropic rearrangement reaction takes place. As per Figure 2(a), the amine group of the probe reacts with FA, and iminium intermediate is formed, which then undergoes 2-aza-Cope sigmatropic rearrangement reaction, which gives rise to a fluorescent product after hydrolysis.

In 2015, Roth et al. [61] had first used this method to detect formaldehyde. They reported a silicon rhodol julolidine-based fluorescent Probe **1** (Figure 2(b)) to detect FA using donor-excited photoinduced electron transfer (d-PET) process [65] in neuro screen-1 and living HEK293TN cells. The detection limit for FA was measured as 0.01 mM (Table 1) in phosphate buffer solution (PBS, pH 7.4), and observed a 3-fold fluorescent increase (excitation: 633 nm) upon the addition of 5 mM FA. Later, Zhao et al. [66] have synthesized a solid portable sensor, Probe **2**, based on aggregation-induced emission (AIE) fluorophore (tetraphenylethylene), for FA detection through fluorescence ‘turn-on’ response (Figure 2(b)). The probe exhibited high selectivity and sensitivity for gaseous FA within 60 min. The sensing limit of the FA test plate was quantified to be 0.036 mg/m^3^ (Table 1). A ratiometric fluorescent FA sensor consisting of 6‐hydroxy naphthalene chromophore (Probe **3)** was developed by Lin and co-workers (Figure 2(b)) [67]. After reaction with an increasing concentration of FA, the emission peak at 451 nm was gradually increased while the peak at 359 nm decreased. Additionally, the probe showed an ability of FA sensing in HeLa cells (Table 1).

**Table 1.** Sensing properties of small molecule-based probes for FA detection

Probe	Fluorophore	Sensing Mechanism	Photophysical Mechanism	Detection Limit	Time	Application	Ref.
1	Silicon rhodol julolidine-based	2-Aza-Cope	Turn-on/PET	0.01 mM	-	Cell imaging (Neuroscreen-1 cellsand live HEK293TN)	[61]
2	Tetraphenyl ethylene	2-Aza-Cope	Turn-on/PET, AIE	0.036 mg/m^3^	60 min	Test plates for gaseousformaldehyde detection	[66]
3	6-Hydroxy naphthalene	2-Aza-Cope	Turn on	-	-	-	[67]
4	BODIPY	Schiff base	Turn on/PET	165 nM	-	-	[71]
5, 6	Benzothiazole & Benzoxazole	Schiff base	Turn-on/PET, ESIPT	2 µM, 29 µM	<3 min	Test strips, cell imaging	[72]
7, 8, 9	Naphthalene	Schiff base	Turn-on/ICT, ESIPT	0.35 µM	100 min	Cell imaging	[73]
10	Benzothiazole- quinoline	Hydrazine	Turn-on/PET	900 nM	-	Food samples and electrospinning test strips	[74]
11	1,8-Naphthalimide	Hydrazine	Turn-on (two photon)/PET	4.9 × 10^−6^ M	40 min	Cell imaging (HeLa cells and mice liver tissues)	[75]
12	1,8-Naphthalimide	Hydrazine	Turn-on/PET	0.36 µM	8 sec	Cell imaging (MCF-7 cells)	[76]
13	Rhodamine B	Aminal	-	3.5 mM	-	Labelling cell surface sialoproteins	[54]
14	Rhodamine 6 G	Aminal	Turn-on	7.7 × 10^−7^ M	-	Dried shiitake mushrooms	[55]

### 2.2. Aromatic amine (Schiff base)-based fluorescent probe

**Figure 3.**
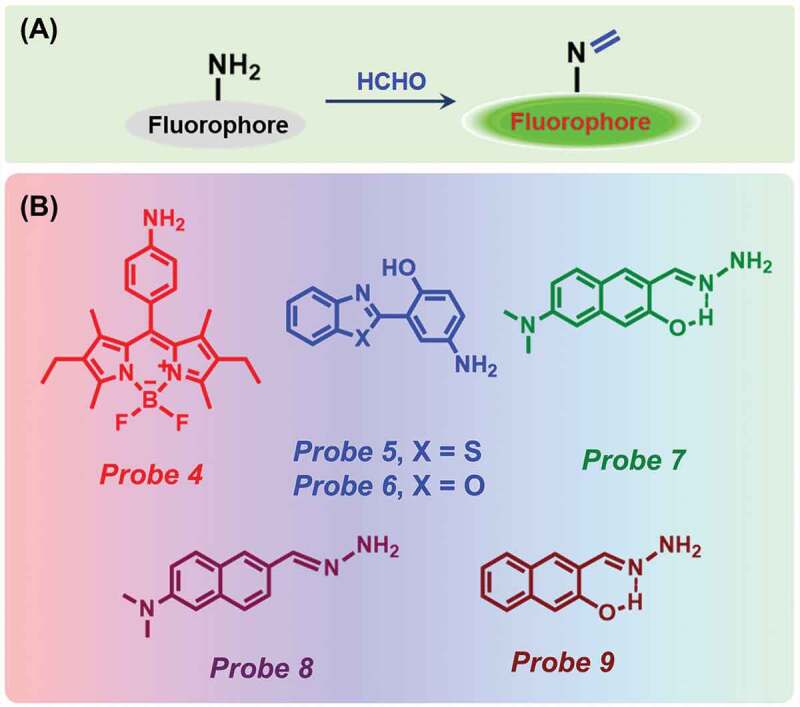
(a) Schematic representation of Schiff base formation reaction, and (b) Chemical structure of different probes.

Schiff bases of the general formula RCH = NR are made by a simple condensation reaction between amine and aldehyde (Figure 3(a)). These reactions are precisely used for generating C-N bonds in organic chemistry with a very high yield [68]. Schiff bases are used as one of the most suitable and useful ligands for the detection of metal ions and some organic molecules due to their strong coordination capability [69,70].

In 2012, Song et al. have used this approach for the first time to selectively detect FA [71]. The difluoroborondipyrromethene (BODIPY)-based Probe **4** (Figure 3(b)) showed a very weak fluorescent peak at 535 nm due to an active PET quenching effect. The effect was quickly demolished after the addition of an increasing amount of FA due to the formation of an imine compound. The detection limit of their probe was estimated as 165 nM (Table 1). Exploiting this method, Wu and co-workers in 2018 have prepared two fluorescent probes composed of benzothiazole (Probe **5**) and benzoxazole (Probe **6**) (Figure 3(b)) unit for the detection of FA utilizing excited state intramolecular proton transfer (ESIPT) method [72]. Probe **5** exhibited two absorption bands at 300 and 365 nm. After the addition of FA, the peak intensity at 365 nm decreased and a 5 nm blue shift was observed. In addition, a significant increase in fluorescence intensity at 519 nm was noticed with a blue shift at 476 nm attributed to the inhibition of PET process between benzothiazole and amino group. The fluorescence quantum yield of the probe was also increased from 0.098 to 0.156. Similarly, for Probe **6**, the addition of FA resulted in an increase of fluorescence intensity at 486 nm along with an enhancement of quantum yield from 0.085 to 0.089. The limit of detection (LOD) of Probes **5** and **6** were determined as 2 and 29 µM, respectively (Table 1). The probes rapidly detected FA in less than 3 min. Besides, test strips were prepared using Probe **5** for quick naked-eye detection of FA. The imaging of FA was successfully achieved in arabidopsis thaliana tissues and MCF-7 cells utilizing Probe **5**.

In a recent work, Chen et al. have fabricated three different naphthalene derivatives (Probes **7, 8** and **9**) as FA sensors (Figure 3(b)) [73]. Among them, only Probe **7** showed high sensitivity and selectivity for FA due to the presence of both ESIPT and ICT effects. After adding FA to the solution of Probe **7 (**PBS buffer solution containing 1% DMSO, pH 7.4), a 14-fold enhancement of emission intensity at 510 nm was observed. The fluorescence enhancement reached a plateau in 100 min upon consecutive addition of 100 μM of FA. The detection limit of Probe **7** for FA was estimated as 0.35 µM (Table 1). On the other hand, Probe **8** displayed less sensitivity toward FA, and in the case of Probe **9**, no change in fluorescence was observed in the presence of FA due to the absence of ICT. Moreover, Probe **7** was effectively used for successful fluorescence imaging of exogenous and endogenous FA in living cells.

### 2.3. Hydrazine-based fluorescent probe

**Figure 4.**
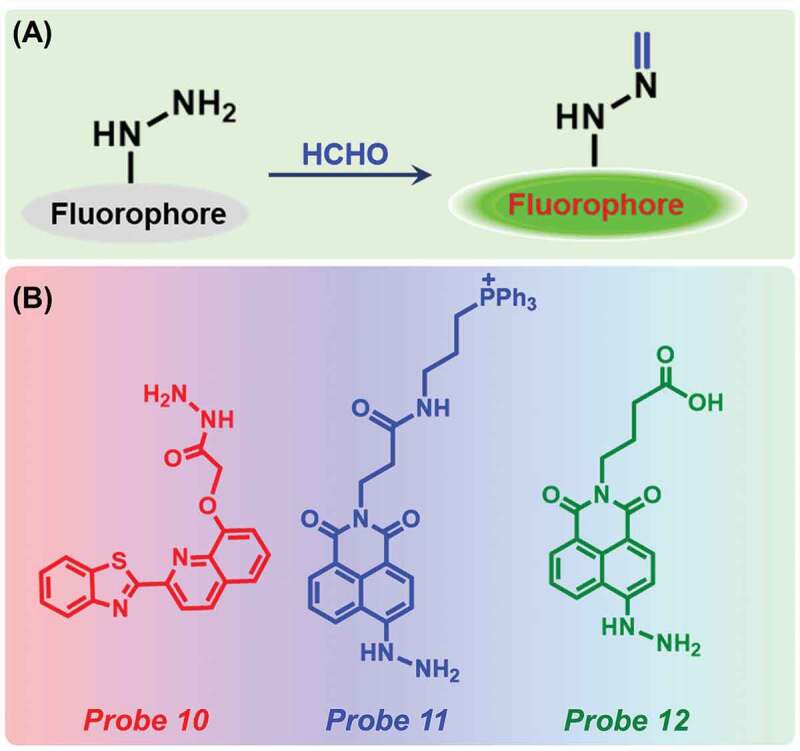
(a) Schematic representation of reaction between hydrazine-based probe with FA and (b) Different sensors for the detection of FA.

In the past few years, several fluorescent probes with hydrazine moiety were developed for selective detection of FA. Hydrazine moiety with an amino group has very strong nucleophilicity which showed high reactivity for FA. Upon reaction with FA, it forms active methylene hydrazine which restricts the PET process from the hydrazine to FA (Figure 4(a)).

Liu et al. have first prepared a hydrazine moiety-based probe **10** (Figure 4(b)) for FA detection [74]. The probe had very weak fluorescence at 467 nm due to PET process from imine to the quinoline fluorophore, however, after adding FA, the fluorescence intensity increased by 5.5-fold due to the elimination of PET effect. The probe showed high selectivity in the presence of several other organic compounds, such as acetone, other aldehydes, formic acid, and acetic acid. The detection limit of the probe was determined as 900 nM (Table 1). The probe was able to successfully detect FA in several food samples. Nanofibers were fabricated by blending the probe with poly(vinyl alcohol) followed by electrospinning. Moreover, upon immersion of the nanofibers into water containing FA, a fluorescence increment was noticed. Later, in 2018, Xu et al. established a two-photon fluorescent probe (Probe **11**, Figure 4(b)) based on hydrazine moiety conjugated 1,8-naphthalimide [75]. Upon addition of FA, the probe displayed a turn-on fluorescence at 539 nm as the condensation reaction of the probe and FA hindered the PET process and the fluorescence intensity reached an equilibrium state at 40 min. The detection limit of Probe **11** was determined to be 4.9 × 10^−6^ M (Table 1). Furthermore, the highly selective probe could be successfully utilized for FA imaging in living mice liver tissues and HeLa cells.

Another naphthalimide-based probe (Probe **12**, Figure 4(b)) was also reported by Yuan and co-workers for the extremely selective and sensitive detection of FA [76]. After the addition of FA to this probe, a blue shift from 449 nm to 435 nm in absorption spectra occurred along with a color change from dark red to bright yellow. The probe by itself displayed very weak fluorescence, however, a substantial increase in fluorescence intensity at 519 nm was observed after the addition of FA, due to the inhibition of PET process. With increasing concentration of FA from 0 to 100 mM, the peak intensity increased linearly. The detection limit was determined as 0.36 µM (Table 1). No changes observed in fluorescence intensity in the presence of several aldehydes (benzaldehyde, *p*-chlorobenzaldehyde, oxalaldehyde, 2,4-dihydroxybenzaldehyde, *p*-hydroxylbenzaldehyde, etc.), metal ions (K^+^, Ca^2+^, Ba^2+^, Na^+^, Mg^2+^, etc.), or other compounds (cysteine, glutathione, homocysteine), indicated high selectivity of the probe. Further, the probe was employed for endogenous FA imaging in MCF-7 cells.

### 2.4. FA detection via aminal formation

**Figure 5.**
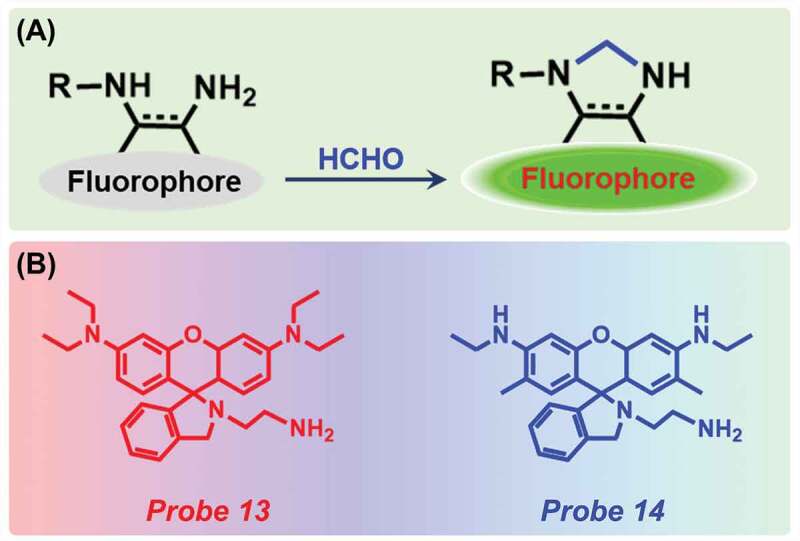
(a) Schematic representation of aminal moiety-based detection technique and (b) probes used for FA detection.

An aminal group is a type of organic compound, having two amine groups attached to the same carbon atom. It has a general chemical formula -C(NR_2_)(NR_2_)-. When FA reacted with this diamine moiety, a condensation reaction took place and an imino intermediate was formed. This intermediate consequently opens the intramolecular deoxylactam, thus forming a strongly fluorescent ring-opened imidazole product (Figure 5(a)).

A rhodamine-based fluorescent probe, *N*-(rhodamine B)-deoxylactam-ethylenediamine (dRBEDA) (Probe **13**, Figure 5(b)) was synthesized by Li et al. for the detection of several aldehydes *via* the formation of aminal intermediate [54]. Interestingly, only with FA, an increase in fluorescence intensity at 560 nm was noticed after 90 min along with the development of red color. The detection limit of the probe was quantified to be 3.5 mM (Table 1). The sensor was capable of labelling cell-surface aldehyde-exhibiting sialoproteins with high selectivity. Later, in 2016, He and co-workers prepared a rhodamine 6 G-based sensor (Probe **14**) for the detection of FA (Figure 5(b)) [55]. The probe was completely nonfluorescent but with the addition of increasing concentrations of FA, the emission peak at 560 nm gradually increased and reached to maximum when 1.0 equivalent FA was present and a color change from colorless to pink was observed. After exciting with 365 nm UV light, naked-eye detection was noticed. The detection limit was estimated as 7.7 × 10^−7^ M (Table 1). To determine the selectivity, the probe was reacted with several other aldehydes, amino acids, glucose but no change was observed. Interestingly, the probe works better in the basic medium than the acidic one. The authors successfully applied the probe for rapid detection of FA in dried shiitake mushrooms.

## 3. Detection of FA using polymeric probes

Most of the small molecular probes are less soluble in aqueous medium which limits their biological applications. There is a need for polymeric sensors with good water solubility, high biocompatibility, low detection limit, and rapid sensing ability. Recently, a lot of organic polymeric probes have been used in the form of coating material, nanoparticles, QCM for the detection of FA.

In 2010, Feng and co-workers reported the first simple yet sensitive formaldehyde detection technique using an amine-terminated polymer Probe **15** (Table 2) [77]. They used regular pH indicators in a polymer film doped into an amine-terminated poly(ethylene glycol) (PEG) polymer which detects FA concentrations of 20 ppm to 250 ppb in less than 1 min and almost 50 ppb of FA within 10 min. Upon interaction of the amine groups present in the polymeric probe with FA, an alteration of pH occurred due to which the indicators showed a colorimetric change. Whereas for other aldehydes almost negligible changes were observed even after exposure for 5 min. This was the first report where FA was detected *via* naked eye.

**Table 2.** Sensing properties of polymeric probes for FA detection

Probe	Polymeric Probe	Detection limit	Detection time	Application	Ref.
15	Amine-terminated PEG	250 ppb	1 min	-	[77]
16	DTA-based probe	1.79 × 10^−8^ M	-	-	[78]
17	Naphthalimide functionalized Chitosan	1.66 µM	<1 min	Sensing in tap water, chicken, bream, and pork	[79]
18	Graphene	10 ppb	-	-	[80]
19	Thiol responsive functionalized silica nanoparticles	36 ppb	-	-	[81]
20	Polyethyleneimine and polyaniline	-	-	Atmospheric FA	[82]
21	Polypyrrole	1 ppm	-	-	[83]
22	PMMA	152 ppb		Indoor air quality	[84]
23	Ethylene glycol dimethacrylate, methacrylic acid, and styrene	500 ppm	-	-	[85]
24	Ethylenediamine (EDA)-functionalized poly(ionic liquid)/polyacrylonitrile	0.036 ppt	-	Home water monitoring	[86]
25, 26	AEMA and PEGMA	3.1 × 10^−7^ and 3.4 × 10^−7^ M	-	Living cells	[87]
27	Polyethyleneimine	50 ppb	100 sec	-	[88]
28	(Fluoral-p) modified polyacrylonitrile	40 ppb	-	-	[89]
29	RGO and PMMA	100 ppm	-	-	[90]
30	Copolymer of primary amine and acrylamide	3 mM	-	-	[91]

**Figure 6.**
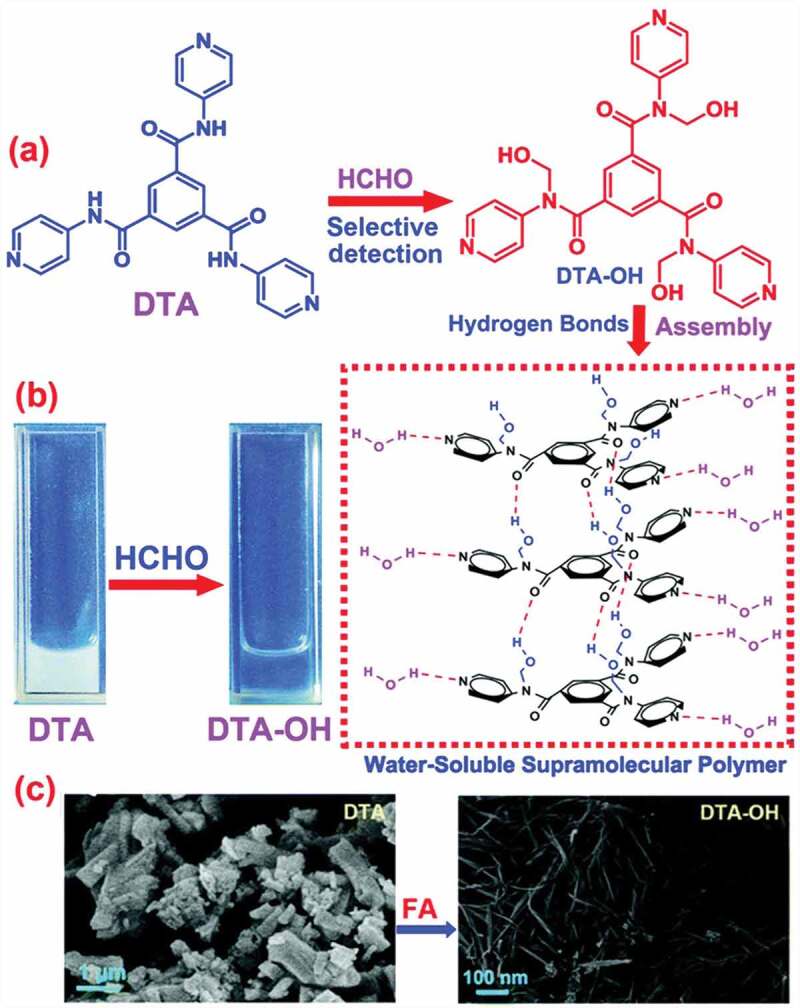
(a) Proposed recognition mechanism of DTA for monitoring FA, (b) photographs of the DTA after addition of FA, and (c) SEM image of DTA before and after FA addition. Reused with permission from ref [78]. Copyright 2019 Royal Society of Chemistry.

In recent years, water-soluble supramolecular polymers have gained significant attention due to their potential biomedical applications. Looking into these aspects for selective detection of FA, Fan and co-workers have reported a water-soluble supramolecular polymer (Probe **16**, Figure 6(a)) using a compound as *N^1^,N^3^,N^5^*-tri(pyridin-4-yl)benzene-1,3,5-tricarboxamide (DTA) [78] (Table 2). The addition of FA solution into DTA resulted in supramolecular self-assembly in water through hydrogen bonding interaction (Figure 6(a)). When several aldehydes were added to DTA, only in the case of FA, the state of DTA changed to a colorless solution and a light blue fluorescence was observed under UV light (*λ*_em_ = 470 nm) (Figure 6(b)). Moreover, a morphological change to fiber structure was noticed after FA addition as confirmed by Scanning Electron Microscopy (SEM) analysis (Figure 6(c)). The LOD of DTA for FA was calculated to be 1.79 × 10^−8^ M.

**Figure 7.**
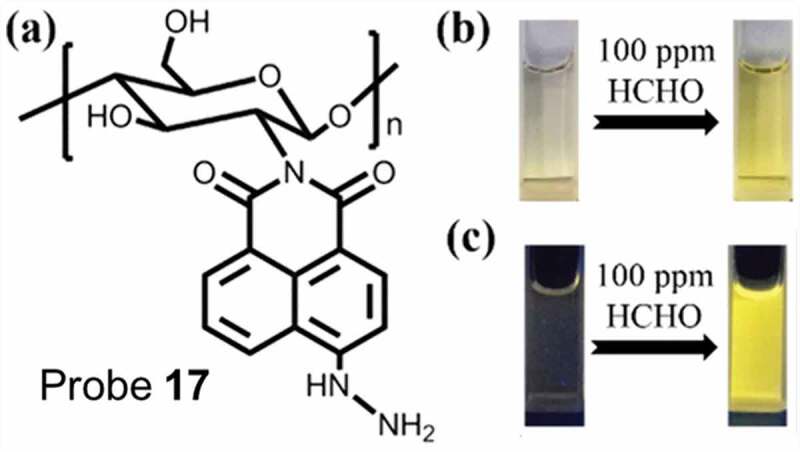
(a) Structure of chitosan-based polymeric sensor. (b) Colorimetric and (c) Fluorometric change of the probe in the presence of 100 ppm FA. Reused with permission from ref [79]. Copyright 2018 American Chemical Society.

Li et al. have reported a naphthalimide functionalized chitosan-based polymeric molecule Probe **17** (Figure 7(a)), where the hydrazine group reacts with FA to generate a turn-on fluorescence [79]. Upon addition of FA, probe **17** showed a colorimetric (colorless to yellow) and fluorometric change (non-emissive to bright yellow) (Figure 7(b) and 7(c)) due to PET process from pendant hydrazine units to naphthalimide fluorophores. Probe **17** rapidly interacted with FA and exhibited a fluorometric alteration within 1 min whereas its small molecular analogue took 30 min to exhibit any notable change. With increasing FA concentration, the emission peak intensities of aqueous solution of Probe **17** gradually increased. The end-to-end hydroxyl group present in the probe enhanced the FA concentration around the polymer chain. In acidic pH range from 1 to 6.5, an improved fluorescence was noticed as acid catalyzed the reaction between FA and naphthalimide groups. The detection limit of the sensor was evaluated to be around 0.05 ppm (1.66 µM) and emission intensity variation was detected with only 1 ppm of FA which further confirmed its sensitivity. Moreover, they successfully applied this sensor to detect HCHO in tap water and three other commercially available food samples as chicken, bream, and pork.

A graphene/poly(methyl methacrylate) (PMMA)-based sensor [80], Probe **18**, was developed by Alizadeh and co-workers for the detection of FA. The device was extremely selective for FA and could sense FA vapor at a precisely small amount and comparatively extensive range of concentration (0.05 and 5.0 ppm). Direct interaction of graphene surface and FA vapor was the main reason for sensor response. The polymeric material acted as an adhesive material when cast on the film. PMMA with more hydrophobic nature effectively deters H_2_O from the polymer surface and therefore from the graphene sheet surface which cuts the sensor response to water molecules. However, FA, which has an additional hydrophobic interaction capability compared to water molecules, can successfully pass over the polymer repulsion wall and result in a signal in the graphene sheet. The limit of detection of this material was calculated to be 10 ppb (Table 2).

**Figure 8.**
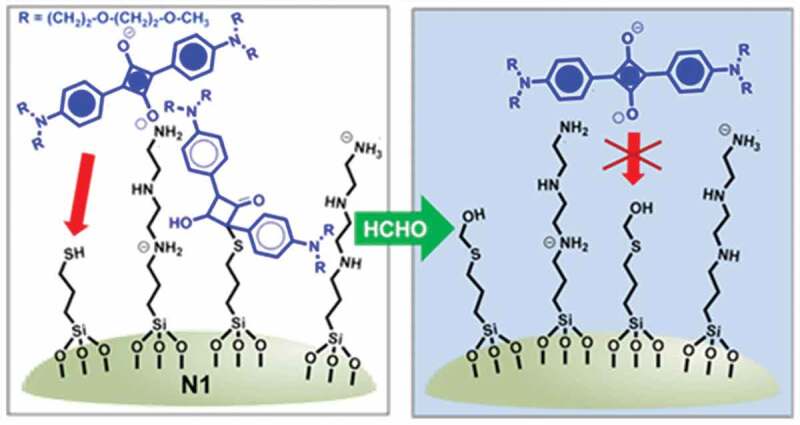
The sensing mechanism of FA using Probe **19**. Reused with permission from ref [81]. Copyright 2016 American Chemical Society.

Sayed and co-workers used a thiol responsive functionalized silica nanoparticle, Probe **19** for the selective colorimetric sensing of FA (Figure 8(a)) [81]. In the probe, the thiol groups interacted with squaraine dye which resulted in a loss of *π*-conjugation property of the chromophore. While, in the presence of FA, this reaction was inhibited and a chromogenic change was observed. Selective response to FA was observed only when both the thiol and polyamine groups were attached to the silica surface. After reaction with FA, a significant enhancement of absorbance at 642 nm was observed along with a colorimetric change from colorless to cyan. The bulky polyamine groups created a highly polar environment around thiols, which only reacted with small and polar FA. Due to the bulkiness of other aldehydes, no change was observed. The LOD for FA was calculated to be 36 ppb in water (Table 2).

Antwi-Boampong and BelBruno have established a polyethyleneimine/polyaniline (PEI/PANi) composite film Probe **20** prepared by spin casting for FA detection [82]. The electrophile carbon atom of FA interacts with the nonbonding electrons on the primary amine functionalities of PEI to reversibly form a polymer bound adduct which is capable of extracting a proton from the doped PANi present in the PEI/PANi composite film. Moreover, chemical interaction with PEI traps FA in the film and increased the probability of interaction with the doped PANi. The film was presented to be receptive to the amount of FA existing in the air. The reaction with FA was expressively better than the response to water vapor, ammonia, or the other volatile organic compounds tested. These results suggest that it is indeed a good detector for real-time FA exposure in atmosphere.

In 2017, Tang and co-workers prepared a sensor Probe **21** with the stability of more than one year for FA detection [83]. A polypyrrole-based molecular imprinted polymer (PPy-based MIP) was combined with a titanium dioxide nanotube (TiO_2_-NTA) to improve the bonding ability of the PPy-based MIP coating. After exposing it to FA, it precisely binds the FA molecules by the hydrogen bond interactions. The attached FA molecules contribute electrons to the PPy-based MIP sheet. The conjugation effect and the swelling effect of the PPy-based MIP layer also accelerate the electron transfer process and increase the conductance. The voids printed on the PPy-based MIP sheet can differentiate FA from other gases by molecular size and monomer functionalities. Apart from that, the sensor shows a decent selectivity for FA than acetic acid, acetaldehyde, acetone, and ethanol, and also very good immunity to humidity. The sensor has a good sensitivity of 13% for 1 ppm FA at room temperature (Table 2).

**Figure 9.**
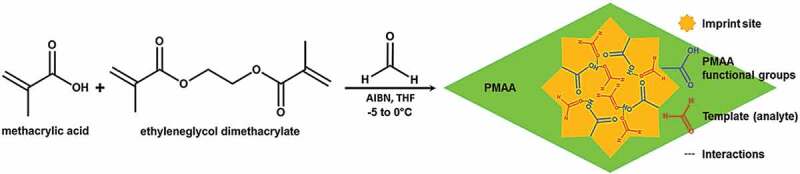
A schematic representation of the low-temperature synthesis of formaldehyde imprinted poly(methacrylic acid) (imp-PMAA). Reused with permission from ref [84]. Copyright 2014 Royal Society of Chemistry.

Iqbal et al. used assemblage of imprinted PMMA and core-shell gold nanoparticles assemblage in layer-by-layer for producing imp-PMAA/Au-NPs hybrid Probe **22** for the sensing study [84]. The procedure for the synthesis of low-temperature imprinted-PMAA is shown in Figure 9. Quartz microbalance (QMB) coated with imp-PMAA/Au-NPs hybrid layer (optimized thickness: 100 ± 20 nm) is used as a transducer. The sensor shows very high sensitivity due to non-covalent dispersion interactions of FA with the molecular recognition sites and enhanced surface area. The sensor has a very low detection limit of 152 ppb and fast response and recovery times, 28 and 13 sec, respectively. Due to its minimal humidity effect, the sensor was used for monitoring indoor air quality.

Another molecularly imprinted polymer (MIP), Probe **23**, was developed by Hussain and co-workers for sensing FA vapors in air [85]. A QCM conjugated with copolymer thin film containing ethylene glycol dimethacrylate, methacrylic acid, and styrene, was prepared. Astonishingly, these MIPs indicated explicit behavior for different Volatile Organic Compounds (VOCs), such as methanol, acetaldehyde, dichloromethane, and formic acid. Although being an apt receptor in principle, the MIPs were not beneficial for amounts at 50% moisture due to surface saturation by water. This was compensated by changing the morphology from thin film to nanoparticles by introducing different primary amino groups into the polymer *via* allylamine. It had a detection limit of 500 ppm (Table 2).

In another study, ethylenediamine (EDA)-functionalized poly(ionic liquid)/polyacrylonitrile nanofibrous membrane (PIL/PAN NFM), Probe **24**, was synthesized as a detector [86]. The poly(ionic liquid) backbones in the ionic microchannels significantly improved electron transportation, thus helped in the detection of FA in aqueous solution by amplifying the current signals. Poly(1-butyl-3-vinyl imidazolium bis(trifluoromethanesulfonyl)imide) ([PBVIm][TFSI]) was used for construction of ionized matrixes and polyacrylonitrile (PAN) for electrospinning. The detection strength was determined as ranging from 360 ppm (3.6 × 10^2^ mg/L) to 0.036 ppt (3.6 × 10^−11^ mg/L) (Table 2). Additionally, the intended ionic microchannels were used for FA detection in real water samples.

**Figure 10.**
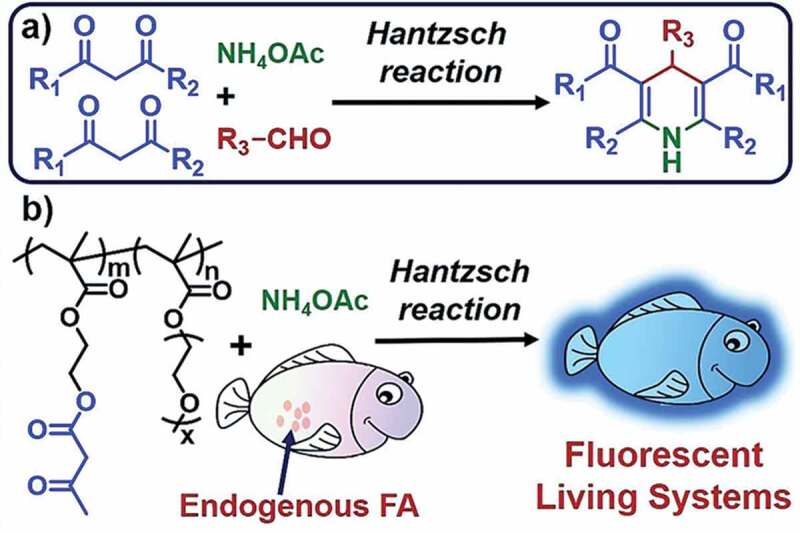
(a) Hantzsch Reaction and (b) the reaction of the polymeric probe with FA. Reused with permission from ref [87]. Copyright 2018 American Chemical Society.

Liu and co-workers have prepared *β*-ketone ester-containing polymers (Probes **25** and **26**) by radical polymerization of 2-(acetoacetoxy)ethyl methacrylate (AEMA) and poly(ethylene glycol methyl ether) methacrylate (PEGMA) in different ratios (for Probe **25** AEMA:PEGMA = 1:1; for Probe **26** AEMA:PEGMA = 1:2) for FA detection *via* Hantzsch reaction (Figure 10(a) and 10(b)) in living systems [87]. Upon incubation of the polymers and their control small molecules with 5 mM FA at 20 C, a strong fluorescence at 460 nm was observed after 5 min in the case of polymers, whereas, even after 30 min the control small molecules had only very weak fluorescence at 510 nm. For the polymeric probes (Probes **25** and **26**), the fluorescent enhancements after 5 min were by 260 and 110 folds respectively, but the control probes had only 21- and 37-folds increase, which proves the significantly high sensitivity of the polymeric probes. The detection limits of Probes **25** and **26** were calculated to be 3.1 × 10^−7^ and 3.4 × 10^−7^ M, respectively (Table 2). Moreover, the polymeric probes successfully detected FA in living cells.

**Figure 11.**
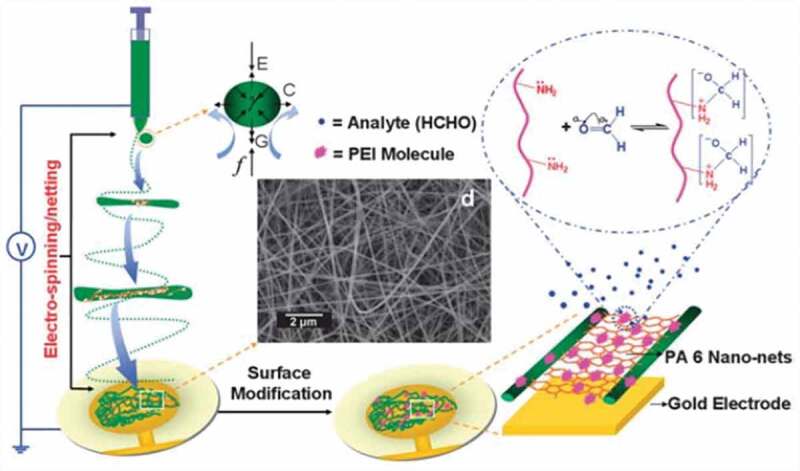
QCM sensing layers schematic representation. Reused with permission from ref [88]. Copyright 2011 Royal Society of Chemistry.

In 2011, Ding and co-workers reported polyethyleneimine (PEI)-functionalized polyamide 6 (PEI-Pa 6) nano-fiber/net (NFN) (Probe **27**), as a new detecting coating material on QCM for extremely sensitive FA detection [88]. The NFN structure (Figure 11) had a big specific surface area, great porosity and large stacking density, which were very useful for sensing. They reported that the detection response mainly happened due to the nucleophilic addition interaction between the amine groups of PEI molecules and FA. Their QCM sensor had a very low detection limit of 50 ppb for FA and a response time of less than 100 seconds (Table 2).

**Figure 12.**
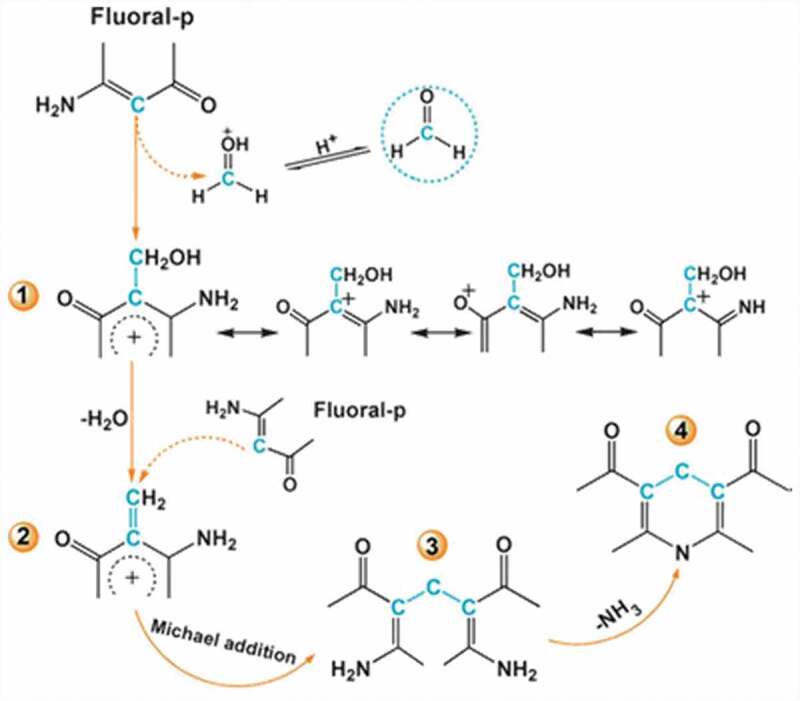
Reaction mechanism of the Probe **28**. Reused with permission from ref [89]. Copyright 2013 Royal Society of Chemistry.

Another sensor strip utilizing 4-amino-3-penten-2-one (fluoral-p) modified electrospun polyacrylonitrile (PAN) (PAN/fluoral-p) (Probe **28**) was developed for naked-eye detection of FA [89]. The sensor strips displayed an important reflectance declining band at 417 nm which brought an intense color change from white to yellow due to the Hantzsch reaction between FA and probe. The probable mechanism of formaldehyde detection is shown in (Figure 12). Due to the enormously big surface area and great porosity of the nanofibrous membranes, the sensitivity is much greater than normal filter paper-based probes. The sensor reached a very low detection limit of 40 ppb.

In 2015, Chuang et al. demonstrated a printable combination material of reduced graphene oxide (RGO) and PMMA Probe **29** for FA sensing [90]. According to their study, 2% RGO/10% PMMA is the ideal ratio for FA sensing. The adsorption of FA disrupts the physical contact between PMMA and RGO, and the sensing sensitivity decreases. The detector exhibited decent selectivity for FA than other VOCs, carbon monoxide (CO), and nitric oxide (NO). The detection level of FA was estimated to be 100 ppm (Table 2).

Kanekiyo and co-workers reported a sensor Probe **30** based on a thin film consisting of primary amino groups on a glass substrate [91]. The FA-responsive thin films were made by the radical copolymerization of a primary amine (*N*-(3-aminopropyl)methacrylamide hydrochloride), acrylamide and cross-linker (*N,N*′-methylene-bis-acrylamide) in the presence of 2,2′-azobis(2-methylpropionamidine)dihydrochloride as an initiator. After exposure with FA, the polymeric thin film was immersed in aqueous anionic dye solutions and the absorption spectra were recorded. They were successful in measuring 0–100 mM FA and the detection limit was calculated to be 3 mM (Table 2). Additionally, the sensitivity of this detector was massively affected by humidity, exposure time, and the monomer conformation of the film.

## 4. Conclusion and future perspective

A systematic review of the development and evolution of the different types of small molecular and polymeric probes for sensitive as well as selective detection of FA, with a special emphasis on the advantages of polymeric probes, is presented here. Through this comprehensive study, we conclude that different types of fluorescent sensors were designed and developed by using the mechanism of aza-Cope rearrangement or FA-amine condensation reaction for extremely selective sensing. Mounting evidence indicates that, among different types of techniques, the reaction-based colorimetric method has emerged as a significant platform for detecting FA, as visible color change appears after selective addition of FA, which in turn is important for real-world applications. Furthermore, various research groups have developed different types of small molecular probes and effectively used them as fluorescent imaging agents for detection of exogenous as well as endogenous formaldehyde in various living animal cells. Apart from the small molecular probes, researchers have explored the potential and advantages of using polymeric probes to overcome the problems of poor aqueous solubility, high detection limit, and longer exposure time, suffered by small molecular probes.

Despite having promising applications, some challenges still exist to further revolutionize this field. A major bottleneck for developing polymeric probes is their synthetic procedures. It is difficult to synthesize large-scale polymeric probes due to the long synthetic pathways. Investigations are underway to overcome these problems to synthesize polymeric materials in industrial scales to detect and mitigate the harmful effects of FA in our daily life. Furthermore, after FA addition, there is always the problem of biodegradability. In this regard, amino acid terminated polymers are evolving as a potential solution. Though developing novel polymeric materials for selective FA detection have gained significant attention of researchers in recent years, only a few reports are available wherein polymeric materials have been successfully used for *in vivo* study.

We believe that a lack of understanding of the reaction mechanism between polymeric probes and FA restricts their full potential. In addition to this, in most of the literature reports, the synthesized sensing systems are for single use only. This limits their applications for environmental monitoring where continuous and repeated monitoring play an important role. Therefore, a thorough investigation of the *in vivo* behavior is the most requirement for their clinical applications and as suitable therapeutic agents. Yet, the finding of novel polymeric materials will offer a ground-breaking push to the quest for new operative treatments for the chronic biological disorders causing damage to a large section of the human population [92]. We are hopeful that this in-depth discussion about FA sensing mechanistic pathway and the recent development of the polymeric probes will highly encourage the scientific community to overcome and find a permanent solution to the FA problem. However, further studies are needed for developing the probable practices of using polymeric materials for successful application in numerous diseases.
